# Factors Associated With Missed Nursing Care in Nursing Homes: A Multicentre Cross-sectional Study

**DOI:** 10.34172/ijhpm.2021.23

**Published:** 2021-04-21

**Authors:** Sara Campagna, Alessio Conti, Marco Clari, Ines Basso, Veronica Sciannameo, Paola Di Giulio, Valerio Dimonte

**Affiliations:** ^1^Department of Public Health and Pediatrics, University of Torino, Torino, Italy.; ^2^Unit of Biostatistics, Epidemiology and Public Health, University of Padova, Padua, Italy.; ^3^Città della Salute e della Scienza di Torino University Hospital, Torino, Italy.

**Keywords:** Missed Nursing Care, Nursing Home, Staffing, Work Factors, Italy

## Abstract

**Background:** Despite its association with patient safety, few studies on missed nursing care have been conducted in nursing homes. We aimed to describe individual and environmental factors in a sample of registered nurses (RNs) reporting missed nursing care in nursing homes, and to explore the association between these factors and missed nursing care.

**Methods:** In the present, multicentre cross-sectional study, 217 RNs from 43 nursing homes in Northern Italy reported all episodes of missed nursing care (ie, any aspect of required care that was omitted or delayed) that occurred in the 20 most dependent residents (according to RNs’ judgement; 860 residents in total) over 3 consecutive days. Multilevel multivariable logistic regression models were used to test possible explanatory factors of missed nursing care (individual, work-related, organisational, and work environment factors), which were entered in a step-wise manner.

**Results:** Younger RNs (*P*=.026), freelance RNs (*P*=.046), RNs with a permanent contract (*P*=.035), and those working in publicly-owned nursing homes reported more episodes of missed nursing care (*P*<.012). Public ownership (odds ratio [OR]=9.88; 95% CI 2.22–44.03; *P*=.003), a higher proportion of residents with severe clinical conditions (OR=2.45; 95% CI 1.12–5.37; *P*=.025), a lower proportion of RNs (OR=2.24; 95% CI 1.10–4.54; *P*=.026), and perceived lack of time to care for residents (OR=2.33; 95% CI 1.04–5.26; *P*=.041) were statistically significantly associated with missed nursing care.

**Conclusion:** Factors associated with missed nursing care are similar in hospitals and nursing homes, and include heavy workload and perceived lack of time for care. Because missed nursing care in nursing homes represents tasks performed specifically by RNs, missed nursing care in this setting should be measured in terms of these tasks. An optimal skill mix is crucial to guarantee not only comfort and basic care for nursing home residents, but also good outcomes for residents with severe clinical conditions.

## Background

**Key Messages**

**Implications for policy makers**

- Policy-makers should be aware that many of the factors that contribute to missed nursing care in the hospital setting, such as heavy workload and perceived lack of time for care, are also relevant in the nursing home setting.
- The number of nursing home residents with more severe clinical conditions is increasing, and nursing home directors should be aware that, without corresponding adjustments in staffing, this increase might lead to missed nursing care.
- Healthcare policies should support a balance in the number of nurse aides and registered nurses (RNs) in nursing homes, in order to ensure both basic and high-level care for residents with severe clinical conditions.

**Implications for the public**

This paper provides recommendations for stakeholders and policy-makers involved in preserving the health and safety of both residents and staff in nursing homes. Laws that outline a minimum level of nurse staffing and the optimal skill mix that should exist in nursing homes need to be introduced to guarantee high-quality care for nursing home residents, more and more of whom have severe clinical conditions.

A growing number of studies from different countries have reported that competing demands and time pressure are latent factors that lead nurses to omit or delay necessary patient care.^1,2^ The level of missed nursing care varies greatly across studies, depending on how the results are reported (mean, composite score, or prevalence), but all studies have shown a consistent effect of this missed care on patient.^3^ Increased episodes of missed nursing care have been associated with worse patient outcomes, including a more negative patient care experience,^4-6^ and an increase in nursing care-related adverse events, such as urinary tract infections, falls, pressure ulcers,^7,8^ and even death.^9^ Missed nursing care also seems to affect nurses’ wellbeing, increasing the occurrence of emotional and physical exhaustion,^10,11^ as well as nurses’ intention to leave their job.^12^ Moreover, registered nurses (RNs) who report more missed nursing care judge the quality and safety of care more negatively.^13^ However, because missed nursing care was only recently defined,^14^ few studies have focused on this phenomenon in nursing homes. The studies that do exist have included samples of nurse aides^15,16^ or both RNs and nurse aides.^17-19^ Only one study examined a sample exclusively composed of RNs; it identified a relationship between burnout, job dissatisfaction, and the occurrence of missed nursing care in nursing homes.^20^

 Missed nursing care in nursing homes varies widely in nature. For example, in studies that included nurse aides, missed nursing care consisted mostly of activities related to basic, hands-on care. In contrast, studies that included RNs reported that missed nursing care was related to documentation, emotional support, monitoring of vital signs, and drug administration. One explanation for this difference might be the distinct responsibilities that these two professional roles comprise, which may lead RNs and nurse aides to judge and report missed nursing care differently. Only one study investigated the severity of missed nursing care in nursing homes, as reported by a sample of RNs; results showed that RNs classified about 25% of missed nursing care as severe due to the clinical consequences and the discomfort caused to residents.^21^

 Missed nursing care has been associated with individual, work-related, organisational, and work environment factors; however, studies on the factors related to missed nursing care in nursing homes are lacking. In a sample of nurse aides, Knopp-Sihota et al found an association between missed nursing care and individual factors (younger age and low level of experience), work-related factors (working on the day shift), organisational factors (low number of beds and public ownership of nursing homes in rural areas), and poor work environment in terms of leadership, culture, staffing, and time for care.^16^ Another study showed an association with the environmental factors high workload and low teamwork perceptions.^19^ Although other organisational factors, such as adequacy of resources and work environment, have been reported to predict the amount of missed nursing care in the hospital setting,^5,22^ staffing and turnover levels did not seem to affect missed nursing care in nursing homes.^19^ Other organisational factors related to missed nursing care in hospitals were RN participation in hospital affairs and collegial nurse-physician relations.^23^

 Knowledge of the factors associated with missed nursing care in nursing homes from the perspective of RNs is important, as RNs not only ensure that nursing home residents are properly assessed and receive the necessary care, they also supervise the activities of nurse aides. Moreover, in order for nursing home directors to prevent or act on missed nursing care, it is crucial to identify the factors associated with these events in the nursing home setting.However,at present there is only limited knowledge on missed care in nursing homes from the perspective of RNs, which means that there is currently no conceptual framework to describe this phenomenon. Therefore, we focused our analyses on factors that have been previously associated with missed nursing care in the hospital setting,^12,24^ ie, individual, work-related, organisational, and work environment factors.The present study aimed to describe these factors in a sample of RNs reporting missed nursing care in nursing homes, and to explore the association between these factors and the occurrence of missed nursing care.

## Methods

 This multicentre, cross-sectional study represents a substudy of a larger project on missed nursing care in nursing homes, which was conducted in the Piedmont Region of Northern Italy between April and October 2018.^21^ Although there are some exceptions, the organisational model and services provided in nursing homes in the Piedmont Region are comparable to those in nursing homes across Italy. Privately- and publicly-owned nursing homes are located throughout the provinces of the Piedmont Region, according to population density, with capacities ranging from 20 to 250 beds. Nursing homes are staffed with RNs and nurse aides: agency staff are usually managed by a social cooperative or recruited directly by the nursing home as independent contractors/freelancers. Permanent-contract workers may also be present in publicly-owned nursing homes. Generally, there are no medical doctors employed at nursing homes; instead general practitioners are responsible for residents’ medical care. The minimum level of nurse staffing required for each resident is set by a multidisciplinary geriatric team, based on residents’ needs. This standard is regulated at a Regional level and is based on a six care levels system (very high, high, medium-high, medium, medium-low, low). Each care level represents an overall minimum standard to be provided, in terms of time spent on care per day per resident.^25^ RNs in nursing homes are responsible for activities like administering therapy, symptoms monitoring, and wound dressings, as well as coordinating nurse aides in performing basic, hands-on care. Residents in nursing homes are usually subdivided into different care units, depending on their care level.

 The present substudy includes RNs from a convenience sample of 43 nursing homes in the Piedmont Region. Two hundred seventeen RNs who were responsible for residents’ care and had more than one month of work experience were asked to report episodes of missed nursing care, defined as any aspect of required patient care that was omitted (either in part or in whole) or delayed,^1^ that occurred in their nursing home. Since nursing homes have different work-related and organisational characteristics, and their residents have clinical conditions of varying severity, participating RNs were asked report episodes of missed care only in the 20 most dependent residents in their respective nursing homes, identified based on RNs’ clinical judgement among those who had a high or very high care level assessed by the multidisciplinary geriatric team. We chose to focus on the most dependent residents because they generally require more nursing care, thus their inclusion would increase the chances of observing episodes of missed care; the number 20 was chosen based on the bed capacity of the nursing homes in the sample, to ensure that each nursing home was equally represented. The 860 identified residents were observed for three consecutive days, starting from an index date between June and July 2018, including day and night shifts performed by RNs.

 During each shift, participating RNs used a specifically designed questionnaire to report omitted or delayed care on previously identified residents. The questionnaire included free-text fields, which RNs used to describe the type of missed care (eg, drug administration, wound dressing change), the cause(s) of missed care (eg, lack of time, lack of resources), when/how the missed care was eventually delivered (duration of delay of care or if care was delegated to another party), whether the missed care was recurrent (ie, had occurred in the in the previous day, week, etc), and the severity of possible consequences for the resident (eg, discomfort and clinical impact, reported on scale of 0–10, with 10 being the most severe). Care that was omitted or delayed due to the resident’s condition (eg, worsening condition) or to adapt a resident’s needs (eg, because the patient was out for a walk or sleeping) were not considered. Each participating RNs described missed care occurred in their individual shift considering each resident they cared for. Descriptive data on missed nursing care are presented in the parent study.^21^

 Individual factors, including RNs’ gender, age, work experience (in their role and in the nursing home), and educational background were assessed. Work-related factors, ie, working shift (day shift vs alternate day and night shift), employment status (agency staff, public/private sector employment, freelancer), and weekly working hours were also considered. Nursing home directors provided work-related and organisational data, including nursing home ownership (private or public) and bed capacity. To estimate workload, the ratio of residents with severe clinical conditions (assessed by the multidisciplinary geriatric team as requiring very high or high care levels) to the total number of occupied beds was calculated. The full-time equivalent (FTE)/100 beds of RNs and nurse aides (full time = 38 hours per week) was used as a measure of staffing, and the skill mix (the ratio of the FTE of RNs to that of nurse aides) was determined.

 Participating RNs completed the nursing home version of the Alberta Context Tool (ACT), which was used to assess work environment. After obtaining permission from the authors of the ACT, the instrument was translated into Italian through a forward-backward translation process, reviewed by a panel of clinical nurses, and its validity was tested in a confirmatory factor analysis. The ACT assesses 10 dimensions of work environment.^26^ Answers are given on a 5-point Likert scale, with higher scores indicating perceptions of a better work environment. The subscales were shown to have good internal consistency, with Cronbach’s alphas ranging between 0.72 and 0.90.^27^ To guarantee privacy and promote free expression, the ACT was completed electronically. Data were treated confidentially.

###  Data Analysis

 Data were examined using R version 3.4.0. The unit of analysis was the individual RN. Median and interquartile range or frequency rates and proportions were described based on continuous or categorical variables. Based on the factors previously identified in the literature,^5,19,22,23^ only five of the ACT dimensions related to work environment were assessed (culture, formal interactions, informal interactions, staffing, and time). Individual, work-related, organisational, and work environment factors were compared between the group of RNs who reported at least one episode of missed nursing care and those who reported no missed nursing care, using the χ^2^ for categorical variables and the Wilcoxon rank-sum test for continuous variables. An overall mean score for each dimension of the ACT was calculated. Odds ratios (ORs) and corresponding 95% confidence intervals (CIs) were obtained by constructing multilevel multivariable logistic regression models, with RNs at level 1 and nursing homes added as a random intercept at level 2. Individual, work-related, organisational, and work environment factors were aggregated at the facility level, and entered in a step-wise manner into the model as potential explanatory variables. Considering the data obtained in our sample, this analytical approach was chosen to focus on the presence of missed nursing care, rather than on the amount of missed nursing care reported. Akaike’s information criterion was calculated to evaluate the goodness of fit of the different models. A lower Akaike’s information criterion indicates a better fit.^28^ The variance inflation factor was calculated with no evidence of multicollinearity among variables (variance inflation factor 0-5).^29^ A two-tailed *P* value of less than.050 was considered statistically significant.

## Results

 Of the 217 included RNs working in the 43 nursing homes in our sample (33 privately owned and 10 publicly owned), 88 (40.5%) reported at least one episode of missed nursing care (mean 0.9 standard deviation = 1.3, range 0-6). The total number of reported episodes of missed nursing care was 189, which affected 150 of the 860 (17.4%) observed residents. More than 1 out of every 6 participating RNs (n = 46; 17%) reported 1 episode of missed nursing care, 7% (n = 20) reported 2, 5% (n = 14) reported 3, and 6% (n = 19) reported more than 3. RNs from 7 nursing homes reported no missed nursing care. Among the 43 nursing homes in the sample, 9 (21.0%) had RNs present for less than 10 hours a day, 16 (37.0%) had RNs present for 10-16 hours a day, and 18 (42.0%) had them for more than 16 hours a day. In 13 (30.2%) nursing homes, a medical doctor was available on site for 3-38 (median 10) hours per week.

 RNs who reported missed nursing care were significantly younger (*P* = .026) (Table 1) and were significantly more likely to be freelance RNs (*P* = .046). Moreover, permanent-contract RNs and those working in publicly-owned nursing homes reported more missed nursing care (40.8% vs 20.4%; *P* = .035 and 30.7% vs 16.3%; *P* = .012, respectively). RNs working in nursing homes with a lower ratio of RNs FTE/100 beds (6.0 vs 6.4; *P* = .001) reported more episodes of missed nursing care, but no association was found for the ratio of nurse aides FTE/100 beds. A lower ratio of RNs to nurse aides (ie, fewer RNs compared to nurse aides) was associated with more reports of missed nursing care (5.8 vs 5.0; *P* = .002). No differences in work environment were observed.

**Table 1:** Individual, Work-Related, Organisational, and Work Environment Factors in the Sample of Participating RNs, and Relationship Between These Factors and Missed Nursing Care

**Factors**	**Respondents**	**Missed Nursing Care**		***P***
	**Total RN (N = 217)**	**Not Reported**	**Reported**	
**Individual**				
Gender^a^ female, No. (%)	170 (81.3)	105 (81.4)	65 (81.2)	.979
Age in years, median (IQR)	30 (26-42)	32 (27-44)	28 (25-38)	**.026**
Work experience in years, median (IQR)				
In role	4 (2.0-12.0)	4 (2.0-16.0)	4 (2.0-8.0)	.121
In nursing homes	4 (1.4-8.0)	4 (1.4-10.0)	3 (1.4-6.0)	.124
Educational background,^a^ No. (%)				.131
Bachelor’s degree	174 (83.7)	104 (80.6)	70 (88.6)	
Vocational diploma	34 (16.3)	25 (19.4)	9 (11.4)	
**Work-related**				
Working shift,^a^ No. (%)				.409
Day shift	110 (52.6)	65 (50.4)	45 (56.2)	
Day and night shifts	99 (47.4)	64 (49.6)	35 (43.8)	
Employment status,^a^ No. (%)				**.046**
Agency staff	86 (41.3)	55 (42.6)	31 (39.2)	
Public/private sector employment	77 (37.0)	53 (41.1)	24 (30.4)	
Freelancers	45 (21.6)	21 (16.3)	24 (30.4)	
Type of contract,^a^ No. (%)				**.035**
Fixed-term	105 (70.5)	76 (76.0)	29 (59.2)	
Permanent-contract	44 (29.5)	24 (24.0)	20 (40.8)	
Weekly working hours, median (IQR)	36.0 (31-38)	36.5 (31-38)	36.0 (31-38)	.863
**Organisational**				
Nursing home ownership, No. (%)				**.012**
Private	169 (77.9)	108 (83.7)	61 (69.3)	
Public	48 (22.1)	21 (16.3)	27 (30.7)	
Bed capacity, No. (%)				.159
<50 beds	50 (23.0)	30 (23.3)	20 (22.7)	
50-100 beds	76 (35.0)	39 (30.2)	37 (42.0)	
>100 beds	91 (41.9)	60 (46.5)	31 (35.2)	
Number of residents with severe clinical conditions/occupied beds	0.3 (0.2-0.6)	0.3 (0.2-0.6)	0.3 (0.2-0.6)	.564
Number of RNs FTE/100 beds	6.4 (5.4-8.5)	6.4 (5.7-9.9)	6.0 (4.6-7.8)	**.001**
Number of Nurse aides FTE/100 beds	37.4 (31.6-41.2)	39.0 (31.6-41.2)	36.7 (30.2-40.7)	.129
Nurse aides/RNs FTE/100 beds	5.5 (4.0-6.5)	5.0 (4.0-6.2)	5.8 (4.6-6.9)	**.002**
**Work environment**				
Culture	3.8 (3.5-4.2)	3.8 (3.5-4.0)	4.0 (3.5-4.2)	.754
Formal interactions	2.5 (2.0-3.2)	2.2 (2.0-3.2)	2.7 (2.0-3.2)	.212
Informal interactions	3.1 (2.6-3.6)	3.1 (2.6-3.6)	3.1 (2.6-3.6)	.949
Staffing	2.7 (2.0-3.7)	2.7 (2.0-3.6)	2.7 (2.0-3.7)	.467
Time	2.2 (2.0-3.0)	2.0 (2.0-3.0)	2.2 (2.0-3.0)	.533

Abbreviations: RNs, registered nurses; IQR, interquartile range; FTE, full time equivalent. ^a^ Presence of missing data.

 Multilevel multivariable logistic regression (Table 2) showed that no individual, work-related, or organisational characteristics were associated with missed nursing care (models 1, 2, and 3). After adjustment for work environment factors (model 4), public ownership, a higher ratio of residents with severe clinical conditions to the number of occupied beds, a lower ratio of RNs to nurse aides, and a perceived lack of time to care for residents were statistically significantly associated with missed nursing care. The probability of reporting missed nursing care in publicly-owned nursing homes was 10 times higher than that in private ones (95% CI 2.22–44.03; *P* = .003). Also, a skill mix with a higher ratio of nurse aides to RNs increased the risk of missed nursing care 2.24 times (95% CI 1.10–4.54; *P* = .026). Lastly, perceived lack of time to care for residents was statistically significantly associated with a higher number of episodes of missed nursing care (OR = 2.33; 95% CI 1.04–5.26; *P* = .041).

**Table 2:** Multilevel Multivariable Logistic Regressions

**Factors**	**Model 1**			**Model 2**			**Model 3**			**Model 4**		
	**OR**	**95% CI**	***P***	**OR**	**95% CI**	***P***	**OR**	**95% CI**	***P***	**OR**	**95% CI**	***P***
**Individual**												
Gender M	1.08	0.43–2.71	.867	0.99	0.39–2.55	.991	0.97	0.36–2.61	.949	1.79	0.56–5.74	.330
Age	0.95	0.88–1.02	.167	0.95	0.88–1.02	.174	0.93	0.86–1.01	.085	0.93	0.85–1.02	.112
Years of experience in role	0.98	0.90–1.08	.697	0.98	0.89–1.07	.643	1.01	0.92–1.11	.830	0.99	0.88–1.11	.805
Bachelor degree vs vocational diploma	0.42	0.07–2.43	.335	0.42	0.07–2.41	.328	0.60	0.11–3.22	.552	0.56	0.08–3.87	.555
**Work-Related**												
Day vs day and night shifts				1.34	0.50–3.60	.565	0.59	0.18–1.91	.377	0.40	0.10–1.57	.190
Freelancers [Ref.]												
Agency staff				0.41	0.12–1.36	.145	0.52	0.16–1.67	.274	0.26	0.06–1.13	.073
Public/private sector employment				0.64	0.18–2.29	.497	1.91	0.47–7.81	.366	1.72	0.33–9.03	.521
Weekly working hours				1.02	0.96–1.08	.616	1.02	0.97–1.08	.437	0.98	0.91–1.05	.511
**Organisational**												
Public vs private ownership							3.25	0.93–11.35	0.065	9.88	2.22–44.03	**.003**
<50 beds [Ref.]												
50-100 beds							1.44	0.38–5.53	.595	4.56	0.76–27.44	.097
>100 beds							0.45	0.11–1.89	.274	2.83	0.42–19.20	.287
Residents with severe clinical conditions/occupied beds							1.78	0.90–3.53	.097	2.45	1.12–5.37	**.025**
Number of RNs FTE/100 beds							1.00	0.77–1.30	.991	1.12	0.83–1.52	.461
Number of Nurse aides FTE/100 beds							0.93	0.82–1.07	.310	0.89	0.75–1.05	.179
Nurse aides/RNs FTE/100 beds							1.71	0.96–3.04	.068	2.24	1.10–4.54	**.026**
**Work Environment**												
Culture										0.93	0.38–2.27	.867
Formal interactions										1.39	0.73–2.64	.317
Informal interactions										1.35	0.61–2.98	.459
Staffing										1.33	0.75–2.38	.334
Time										2.33	1.04–5.26	**.041 **
**AIC**	259.757			261.108			252.266			202.487		

Abbreviations: OR, odds ratio; CI, confidence interval; M, male; Ref, reference; RN, registered nurse; FTE, full-time equivalent; AIC, Akaike’s information criterion.

## Discussion

 In this multicentre study of 43 Italian nursing homes, we examined the relationship between an extensive set of individual, work-related, organisational, and work environment factors and the likelihood that RNs will omit or delay nursing care. We observed that many of the factors that contribute to missed nursing care in the hospital setting are also relevant in the nursing home setting. One of the most relevant results of our study was that a higher proportion of residents with severe clinical conditions affects missed nursing care. This is particularly relevant given the increasing number of nursing home residents with severe clinical conditions,^30^ as well as those with behavioural and psychological conditions like dementia.^31^ Indeed, these residents require specific healthcare services, and the delivery of those services is coordinated mostly by RNs.^32^ In light of this, nursing home directors must carefully consider the proportion of residents with severe clinical conditions in their facility in order to hire a skill mix of personnel that can provide proper care. Often, RNs are replaced with less skilled personnel to reduce staff costs, and because nursing home directors have difficulties recruiting qualified employees.^33^

 Our findings support previous results that showed no benefits to increasing the number of nurse aides at the expense of RNs.^12,34,35^ In nursing homes, as in others healthcare institutions, nurse aides spend most of their time providing hands-on care and performing other basic tasks,^36^ whereas RNs provide care that requires a higher skill level, such as setting up or updating residents’ care plans, providing emotional support, managing social or rehabilitation care, and administering drugs.^19,21^ These activities exceed the scope of care that can be performed by nurse aides, and thus can’t be delegated. Previous studies showed that nurse aides frequently reported missed nursing care that comprised activities consistent with their responsibilities, ranging from monitoring of vital signs to taking residents for a walk.^15,37^ Therefore, separate assessments of missed care should be conducted for RNs and nurse aides, as the nature of this care seems to be related to the role and responsibilities of their respective professions. Moreover, staffing measures have been weakly correlated with patient outcomes,^38^ and further research is needed to identify the explanatory power of these measures with regard to missed nursing care.

 In agreement with the findings of Henderson et al, our study highlighted that RNs working in a publicly-owned nursing homes reported more missed nursing care than those working in privately-owned facilities.^39^ It is plausible that work-related and organisational factors differ between publicly- and privately-owned nursing homes. Previous studies have found a lower standard of care within the private sector,^40^ also because publicly-owned facilities tend to be better staffed.^39^ Moreover, we found that freelance RNs and RNs with a permanent contract were more likely to report missed nursing care. This could be due to barriers to reporting missed nursing care, such as fear of disciplinary action – including getting fired – and lack of confidentiality due to the small number of RNs working in each nursing home.^41^

 With regard to work environment factors, only perceived lack of time to care for residents was associated with missed nursing care. This is consistent with previous research in nursing homes, which found that a perceived high workload is linked to missed nursing care.^16,19^ Time constraints may lead RNs to prioritise the monitoring of symptoms and the detection of changes in residents’ health status, as these tasks are necessary to correctly plan residents’ care, and because missing these tasks could lead to negative resident outcomes. But this prioritisation means that RNs may choose to leave documentation or emotional support undone.^19^ Interestingly, perceived lack of time to care for residents, staffing level (number of nurse aides/RNs FTE/100 beds), and the presence of residents with severe clinical conditions are significantly associated with each other. The perception of increased workload can be influenced by personnel issues and increases in scheduled and unscheduled work activity, such as staff turnover, a new admission, or a resident’s sudden worsening or death.^19^

 Our results underline the importance of striking the optimal balance between RNs and nurse aides in order to guarantee not only comfort and basic care, but a high quality of care for nursing home residents with severe clinical conditions.^42^ Unfortunately, cost conservation appears to be the predominant concern of nursing home directors, and nurse aide salaries are lower than those of RNs. However, although it is clear that nurse aides provide the majority of basic, hands-on care to nursing home residents and play an essential role in long-term care, further investments towards the recruitment of RNs could also save costs by limiting emergency department use and hospitalisations.^43^ Indeed, RNs play a pivotal role in the early recognition of common clinical situations that occur in nursing homes, such as urinary tract and pulmonary infections, iatrogenic complications, gastroenteritis, and heart failure.^7,8^

 Nursing homes directors should take into account RNs’ perceived workload, as it is significantly related to missed nursing care. Objective data alone cannot always capture the changing nature of working conditions in nursing homes, such as the quick worsening of patients’ health status and sudden increases in workload. Thus, policy-makers should be aware of problems related to the fluctuation of job demands that follow sudden changes in residents’ conditions, high rates of admittance and discharge, and unexpected organisational demands. Directors of privately-owned nursing homes should pay particular attention to these aspects to guarantee working conditions that are the same as those in publicly-owned facilities. Few countries have introduced minimal nurse staffing laws or required a 24-hour RN presence in nursing homes. This is surprising, as a recent study determined that an additional two minutes of registered nursing care can improve residents’ outcomes by up to 8%.^44^ The current challenge when proposing new health policies is to establish a set of evidence-based criteria that takes into account residents’ clinical severity and contextual characteristics to determine an appropriate skill mix and address the multi-faceted needs of nursing home residents, thus ensuring the highest standards of care.

###  Strengths and Limitations

 This study contributes to the assessment of understudied factors that affect missed nursing care among RNs in nursing homes. The adoption of a cross-sectional design did not allow us to show causality between variables. Despite this, the availability of administrative data, data collection from nursing home directors and RNs, and the prospective, direct reporting of missed nursing care (instead of retrospectively filled tools) allowed us to prospectively assess missed nursing care and to reflect on what RNs classify as missed nursing care. Furthermore, multilevel multivariable logistic regression models took into account the most important individual, work-related, organisational, and work environment factors. Nonetheless, some limitations should be taken into account. First, although data were collected anonymously, RNs may not have reported some omissions or delays in care due to social desirability concerns or recall bias. Second, given the Piedmont Region context, characterised by publicly- and privately-owned nursing homes that have a wide variation in capacity (from 20 to 250 beds) and where staff are generally managed by a social cooperative or hired by the nursing home as freelancers or independent contractors, the generalisability of these findings should be considered with caution. Lastly, we only considered five of the 10 ACT dimensions in the analyses, which may have limited our ability to identify work environment factors associated with missed nursing care. Social capital describes the stock of active connections among people; however, the inclusion of formal and informal interactions in the analyses did help capture some of aspects of teamwork and communication.

## Conclusion and Implications

 Missed nursing care is a phenomenon that is present in all care contexts, including nursing homes. Indeed, the present study showed that many of the factors associated with missed nursing care in hospitals are also relevant in nursing homes, such as the presence of patients with severe clinical conditions, less qualified staff, and a higher perceived workload. The increasing proportion of nursing home residents with severe clinical conditions should be considered in the hiring and management of RNs, as this factor was associated with increased episodes of missed nursing care. Nursing homes are increasingly becoming the places where people live until the end of their life. Thus, it is fundamental to ensure residents’ safety, provide a high quality of care, effective communication, and be respectful of each resident’s choices. Given the association between a lower ratio of RNs to nurse aides and increased episodes of missed nursing care, efforts should be made to discourage the replacement of qualified employees with scarcely qualified and unprepared personnel. RNs can be a key resource for improving the quality of care offered in nursing homes. Thus, it is essential that nursing home directors introduce measures targeted at reducing RNs’ perceived workload and improve organisational processes to foster more responsible time management.

## Acknowledgements

 We are grateful to the nursing homes staff that took part in this study, and to the Piedmont Regional Nursing Council for the support in recruitment of the Nursing Homes.

## Ethical issues

 Ethical approval was obtained by an independent bioethics committee (University of Torino Bioethics Committee - Prot. 291041 of 13/07/2018).

## Competing interests

 Authors declare that they have no competing interests.

## Authors’ contributions

 SC, PDG, and VD were responsible for the study concept and design. IB and AC were responsible for the data acquisition. VS, SC, MC, PDG, and VD analysed and interpreted the collected data. SC, AC, MC, IB, and VS were responsible for writing the report. PDG and VD provided critical revision of the manuscript for relevant intellectual content.

## Funding

 This research received a specific grant from the Piedmont Regional Nursing Council (Coordinamento Regionale degli Ordini delle Professioni Infermieristiche). The Piedmont Regional Nursing Council had no role in the study design; collection, analysis, and interpretation of data; written of the manuscript; or decision to submit the manuscript for publication.
